# Supplementary material for: De novo sensorimotor learning through reuse of movement components
Source: PLoS Comput Biol. 2024 Oct 10;20(10):e1012492. doi: 10.1371/journal.pcbi.1012492 (PMC11495618; doi:10.1371/journal.pcbi.1012492)
Supplement: S1 Fig — Alignment of the peak of the observed cursor trajectory with the peak of the target trajectory results in more target hits. Throughout the reported analyses, we use the peak-aligned trajectories to compute metrics of trajectory shape quality. (DOCX) [file pcbi.1012492.s001.docx]

## Supplementary Figures

| 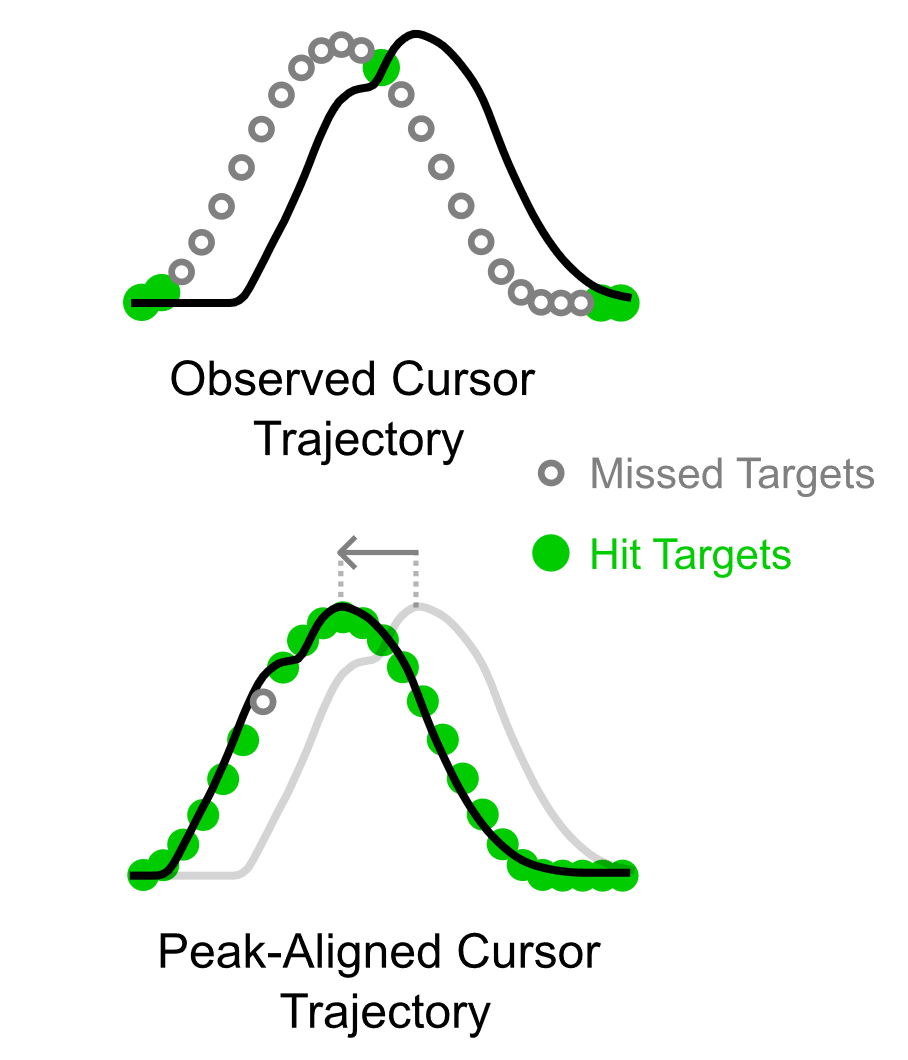 |
| --- |
| ***S1 Fig – Demonstration of trajectory peak alignment.*** *Alignment of the peak of the observed cursor trajectory with the peak of the target trajectory results in more target hits. Throughout the reported analyses, we use the peak-aligned trajectories to compute metrics of trajectory shape quality.* |
