# Supplementary material for: De novo sensorimotor learning through reuse of movement components
Source: PLoS Comput Biol. 2024 Oct 10;20(10):e1012492. doi: 10.1371/journal.pcbi.1012492 (PMC11495618; doi:10.1371/journal.pcbi.1012492)
Supplement: S2 Fig — A) KDE approximated distributions of observed per-channel peak amplitudes in session 5 for two example participants (C4, congruent; I8 incongruent), separated by trial condition. Vertical lines indicate the modes of each distribution. The distributions for these participants are clearly distinguishable. B) Distributions as in A, but for two other participants (C6 congruent, I2 incongruent) showing extensive overlap for the three trial conditions. C) All participants were assigned to one of two groups based on whether the peak amplitude distributions in at least one of the two channels were distinguishable. Participants were assigned to the “distinguishable” group (N = 7; 3 congruent) if the modal amplitudes for the three conditions were all more than 0.2 apart and assigned to the “indistinguishable” group otherwise (N = 11; 6 congruent). Plots show posterior Log-RMS marginalised across all participants in each distinguishable/indistinguishable group (purple points and lines). Red points are marginal posterior means for participants in the incongruent group, blue for the congruent group. Inset Bayes factors are in favour of a reduction from session 1 to session 5. D) Similar to C, but for trajectory peak time. Participants I3 and C8 are excluded from all analyses in this figure. (DOCX) [file pcbi.1012492.s002.docx]

| 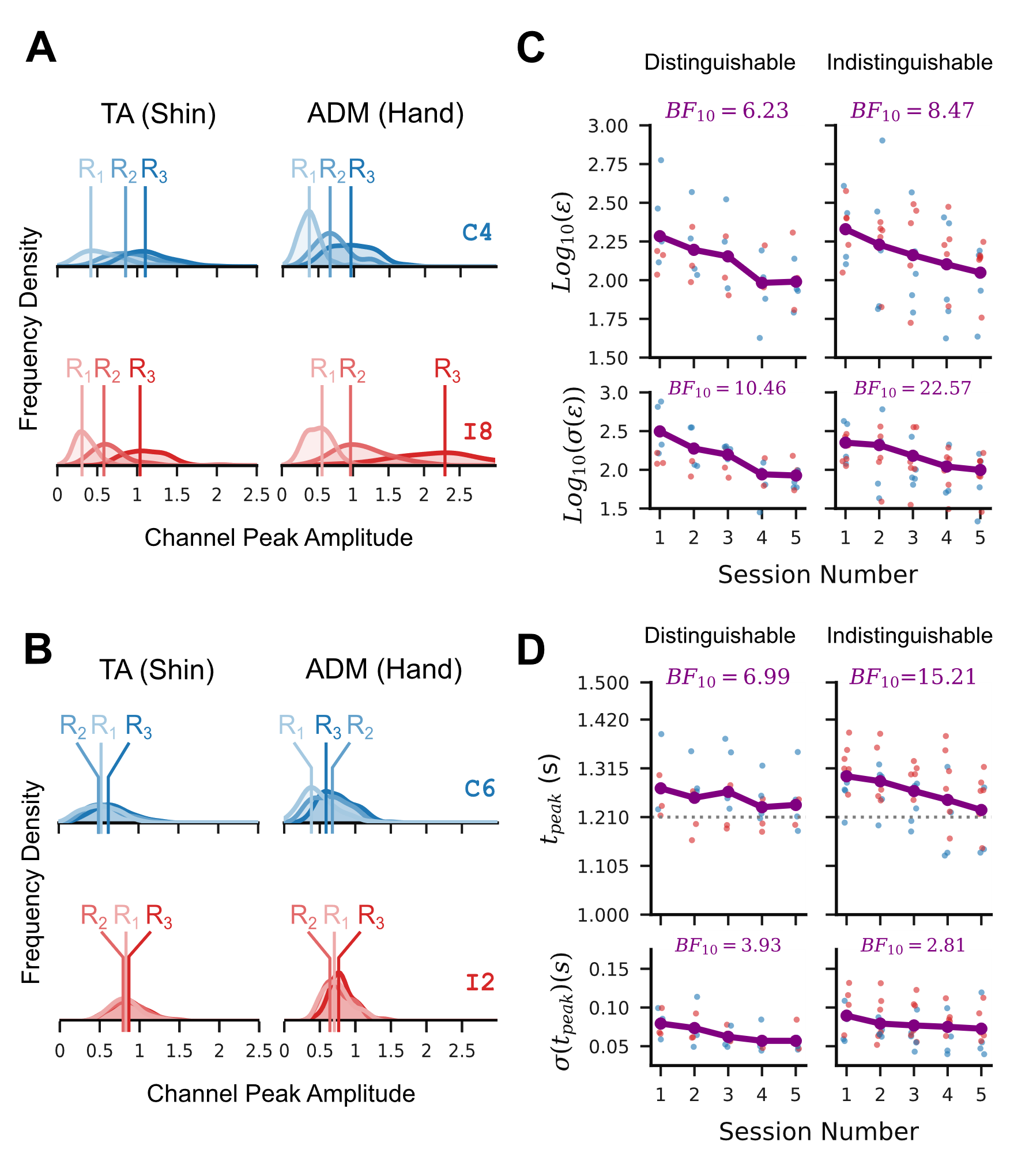 |
| --- |
| ***S2 Fig – Participants applied untrained condition-specific outputs or re-used existing non-specific outputs to achieve transfer of performance gains to the untrained R_2_ condition****. A) KDE approximated distributions of observed per-channel peak amplitudes in session 5 for two example participants (C4, congruent; I8 incongruent), separated by trial condition. Vertical lines indicate the modes of each distribution. The distributions for these participants are clearly distinguishable. B) Distributions as in A, but for two other participants (C6 congruent, I2 incongruent) showing extensive overlap for the three trial conditions. C) All participants were assigned to one of two groups based on whether the peak amplitude distributions in at least one of the two channels were distinguishable. Participants were assigned to the “distinguishable” group (N = 7; 3 congruent) if the modal amplitudes for the three conditions were all more than 0.2 apart and assigned to the “indistinguishable” group otherwise (N = 11; 6 congruent). Plots show posterior Log-RMS marginalised across all participants in each distinguishable/indistinguishable group (purple points and lines). Red points are marginal posterior means for participants in the incongruent group, blue for the congruent group. Inset Bayes factors are in favour of a reduction from session 1 to session 5. D) Similar to C, but for trajectory peak time. Participants I3 and C8 are excluded from all analyses in this figure.* |
