# Supplementary material for: De novo sensorimotor learning through reuse of movement components
Source: PLoS Comput Biol. 2024 Oct 10;20(10):e1012492. doi: 10.1371/journal.pcbi.1012492 (PMC11495618; doi:10.1371/journal.pcbi.1012492)
Supplement: S3 Fig — (A) Marginal posterior distributions for the difference in log-RMS between the rightward and leftward “no-feedback” trials. Individual horizontal lines are per-participant 95% posterior credible intervals. Shaded curves represent posterior density of the difference across all participants. Red features represent participants from the incongruent group, blue features represent participants from the congruent group. Columns correspond to different trajectory magnitudes. (B) Marginal posterior distributions for the difference in trajectory peak time between the rightward and leftward “no-feedback” trials. Features are as in A. (DOCX) [file pcbi.1012492.s003.docx]

| 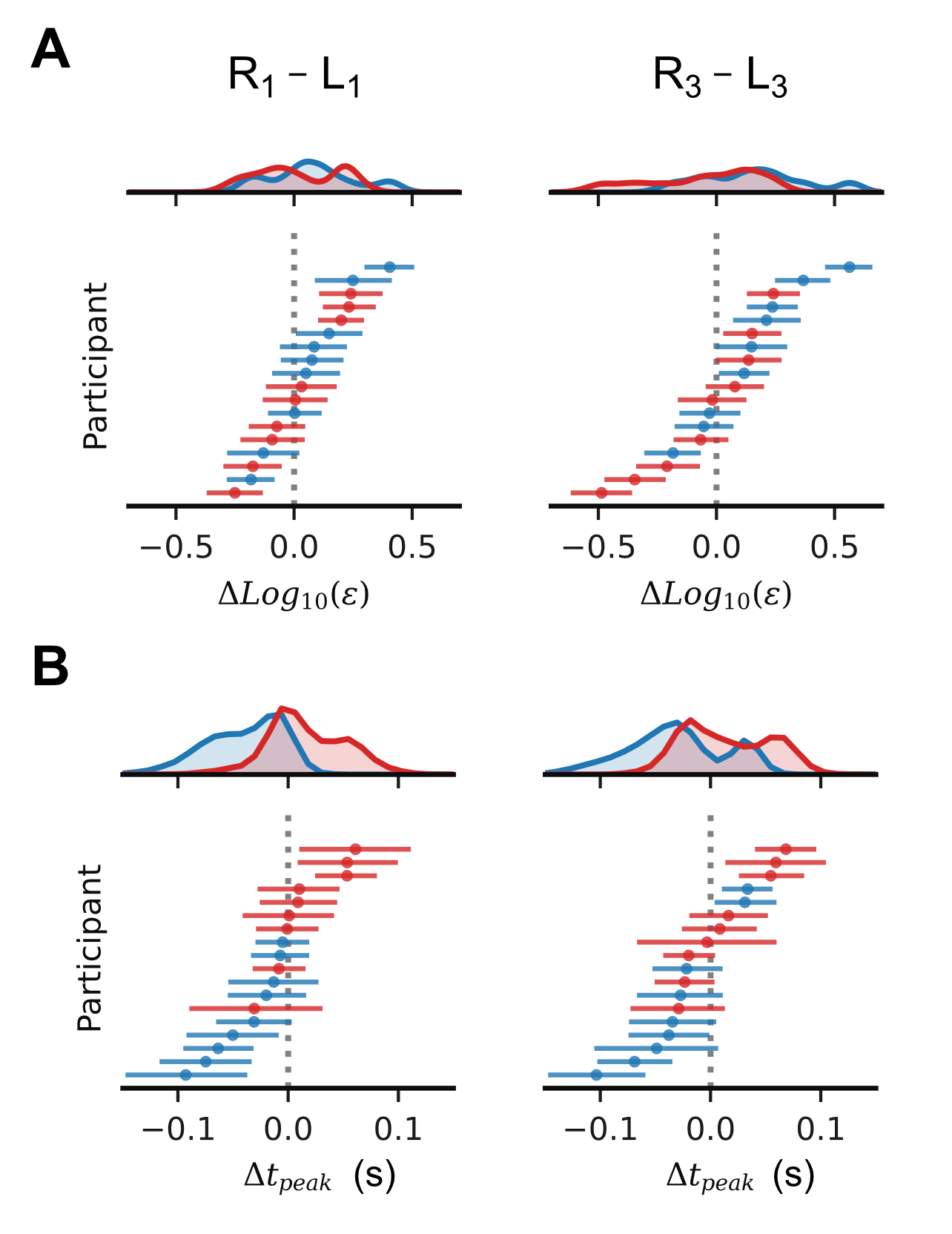 |
| --- |
| ***S3 Fig – Participants had idiosyncratic biases in error across the two trajectory directions, but neither the rightward nor leftward paths were intrinsically more difficult****. (A) Marginal posterior distributions for the difference in log-RMS between the rightward and leftward “no-feedback” trials. Individual horizontal lines are per-participant 95% posterior credible intervals. Shaded curves represent posterior density of the difference across all participants. Red features represent participants from the incongruent group, blue features represent participants from the congruent group. Columns correspond to different trajectory magnitudes. (B) Marginal posterior distributions for the difference in trajectory peak time between the rightward and leftward “no-feedback” trials. Features are as in A.* |
