# Supplementary material for: De novo sensorimotor learning through reuse of movement components
Source: PLoS Comput Biol. 2024 Oct 10;20(10):e1012492. doi: 10.1371/journal.pcbi.1012492 (PMC11495618; doi:10.1371/journal.pcbi.1012492)
Supplement: S4 Fig — Plots show the mean peak-aligned per-participant channel profiles in R3 trials (dark lines) and L3 test trials in session 5. Dotted lines indicate the mean peak amplitudes for the R3 trials. Red traces correspond to incongruent group participants, and blue traces correspond to congruent group participants. Results for other sessions and path magnitudes are qualitatively similar. (DOCX) [file pcbi.1012492.s004.docx]

| 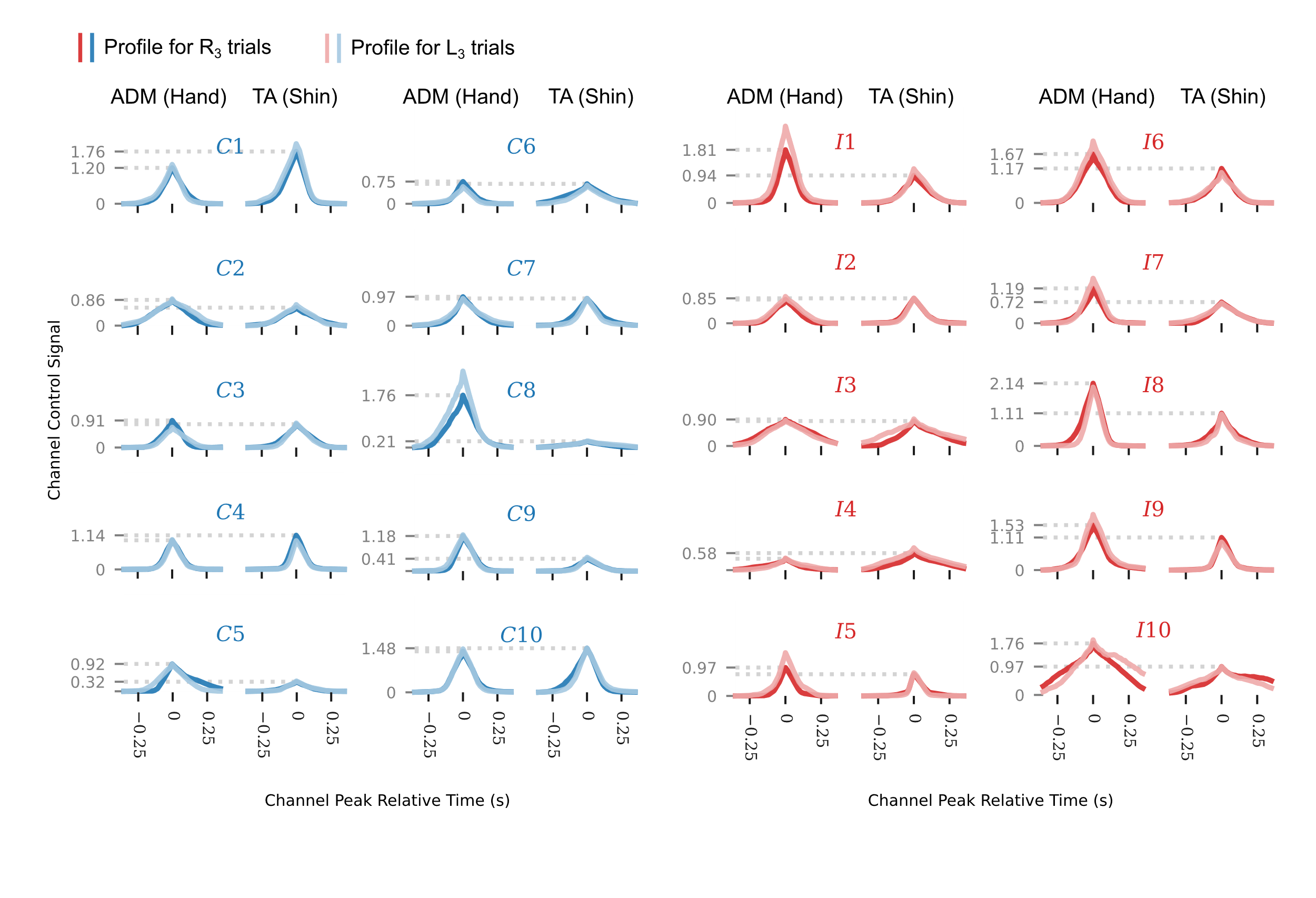 |
| --- |
| ***S4 Fig – Per-participant mean channel profiles were re-used across trajectory directions.*** *Plots show the mean peak-aligned per-participant channel profiles in R_3_ trials (dark lines) and L_3_ test trials in session 5. Dotted lines indicate the mean peak amplitudes for the R_3_ trials. Red traces correspond to incongruent group participants, and blue traces correspond to congruent group participants. Results for other sessions and path magnitudes are qualitatively similar.* |
