# Supplementary material for: De novo sensorimotor learning through reuse of movement components
Source: PLoS Comput Biol. 2024 Oct 10;20(10):e1012492. doi: 10.1371/journal.pcbi.1012492 (PMC11495618; doi:10.1371/journal.pcbi.1012492)
Supplement: S6 Fig — (A) Points show the channel peak amplitude of a selected R2 or L2 trial from session 5 plotted against that of a trial of any condition from session 1. The trials were selected by computing the minimum difference between the channel amplitude of a given trial in session 5 and the channel amplitudes of all trials in session 1, then selecting from these minimums the pair whose magnitude difference was the 99th percentile value. (B) Similar to A, but for per channel peak time. (DOCX) [file pcbi.1012492.s006.docx]

| 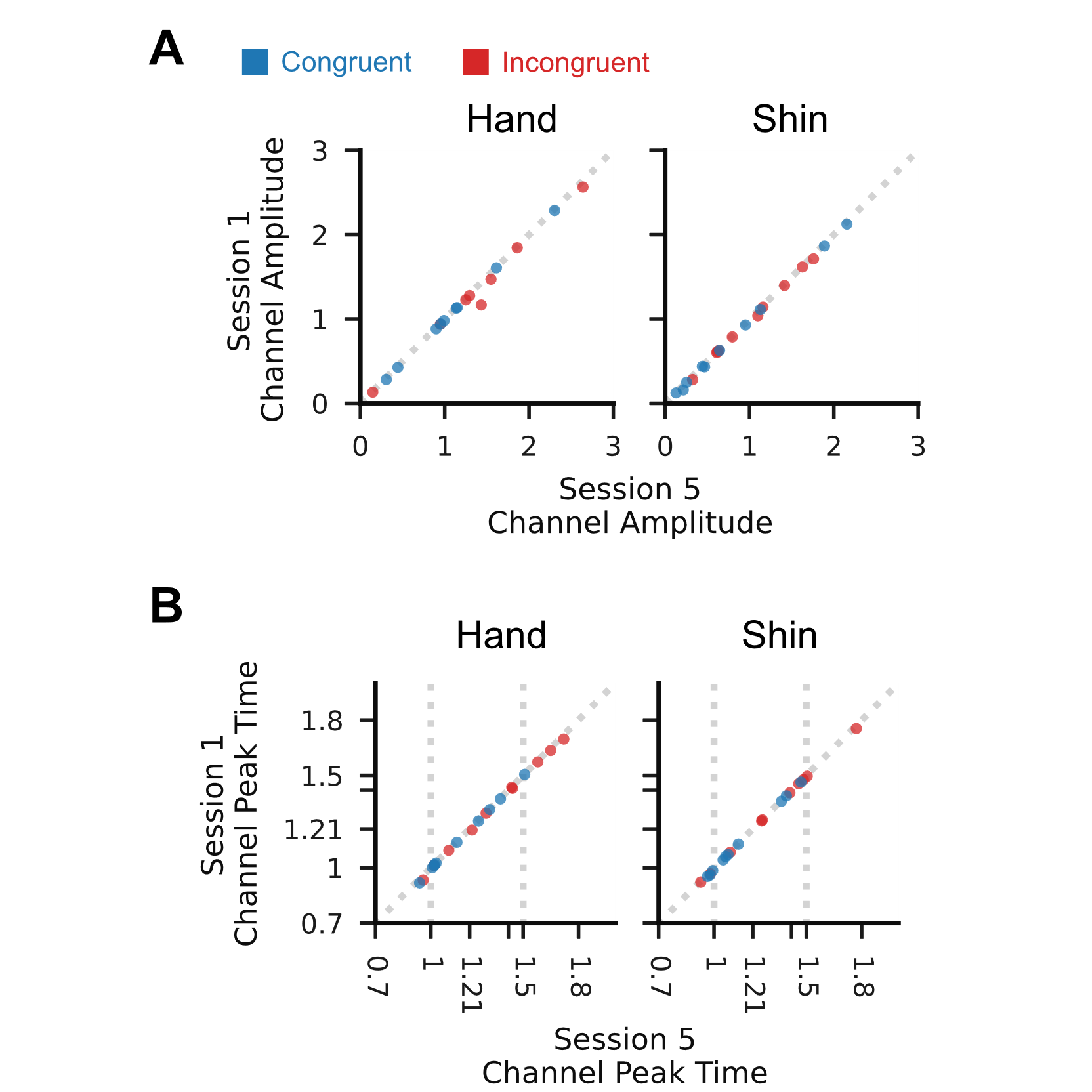 |
| --- |
| ***S6 Fig – Participants could produce per-channel outputs for R_2_ and L_2_ in session 1 which closely resembled those used in session 5.*** *(A) Points show the channel peak amplitude of a selected R_2_ or L_2_ trial from session 5 plotted against that of a trial of any condition from session 1. The trials were selected by computing the minimum difference between the channel amplitude of a given trial in session 5 and the channel amplitudes of all trials in session 1, then selecting from these minimums the pair whose magnitude difference was the 99^th^ percentile value. (B) Similar to A, but for per channel peak time.* |
