# Supplementary material for: De novo sensorimotor learning through reuse of movement components
Source: PLoS Comput Biol. 2024 Oct 10;20(10):e1012492. doi: 10.1371/journal.pcbi.1012492 (PMC11495618; doi:10.1371/journal.pcbi.1012492)
Supplement: S7 Fig — (A) Upper plots show the top three functional principal components of the hand channel profiles, computed using the hand profiles from all no-feedback trials in which the order of hand and shin channel peaks was correct. These three components are sufficient to explain 89.3% of the variance in generated hand channel profiles across all sessions and participants. Each generated channel profile can therefore be well approximated by a linear combination of these three features. We project each participant’s hand channel profiles onto each component to get a three-dimensional representation of the hand channel profiles generated on each trial. We then assess the similarity of these components in session 5 and session 1, as explained in S6 and S7 Figs. In this case, we simultaneously compare all six path conditions in session 5 to all six conditions in session 1. As before, we observe that, for 99% of the trials, the generated hand channel profile in session 5 is very similar (in fPCA feature space) to a hand channel profile generated during session 1. (B) Analyses are as in A, but using the shin channel profile. The three selected fPCA components explained 85.8% of the variance in shin channel profiles across all participants and no-feedback trial conditions. (DOCX) [file pcbi.1012492.s007.docx]

| 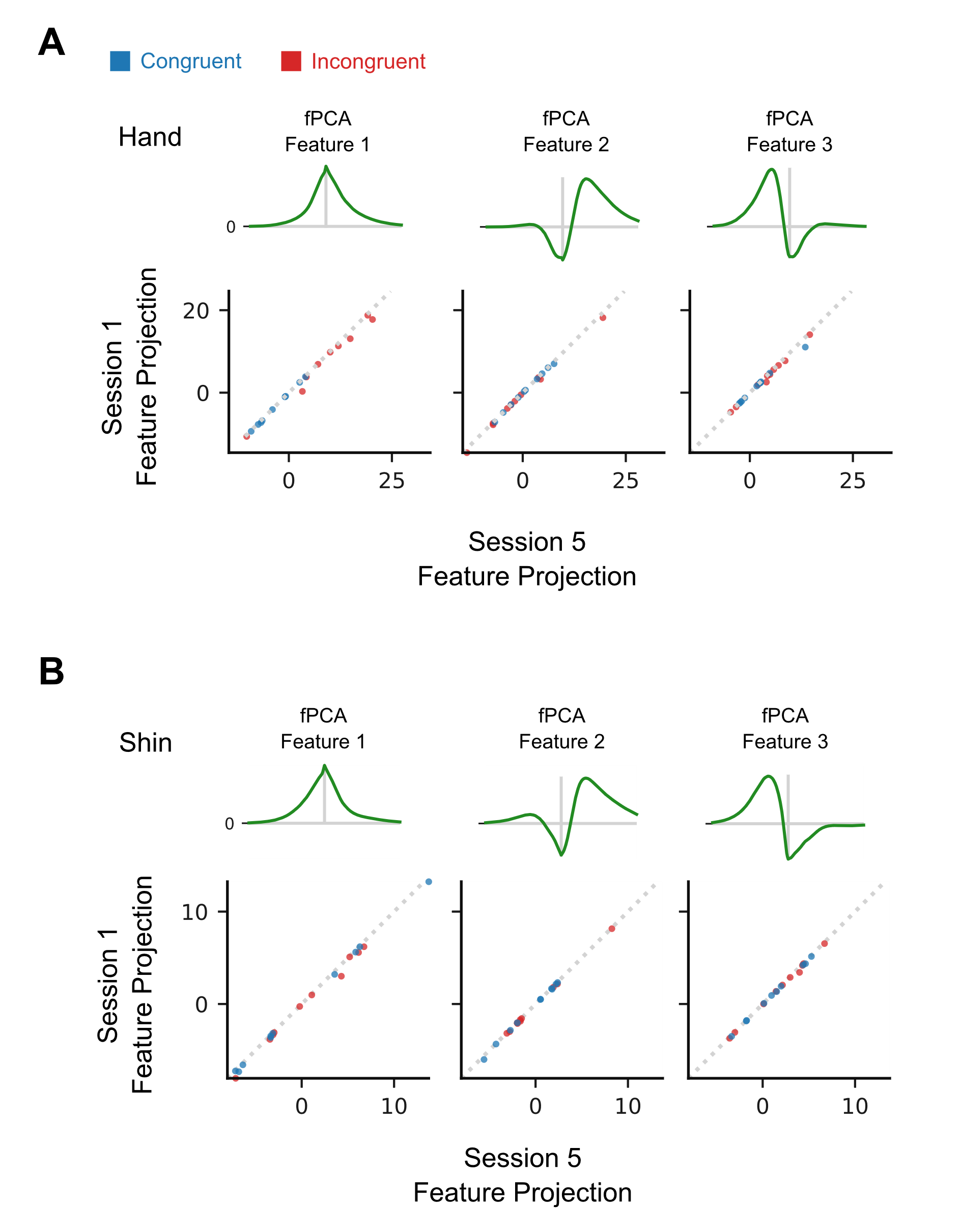 |
| --- |
| ***S7 Fig – Participants could produce per-channel outputs for all path shapes in session 1 which closely resembled those used in session 5.*** *(A) Upper plots show the top three functional principal components of the hand channel profiles, computed using the hand profiles from all no-feedback trials in which the order of hand and shin channel peaks was correct. These three components are sufficient to explain 89.3% of the variance in generated hand channel profiles across all sessions and participants. Each generated channel profile can therefore be well approximated by a linear combination of these three features. We project each participant’s hand channel profiles onto each component to get a three-dimensional representation of the hand channel profiles generated on each trial. We then assess the similarity of these components in session 5 and session 1, as explained in Figures S6-7. In this case, we simultaneously compare all six path conditions in session 5 to all six conditions in session 1. As before, we observe that, for 99% of the trials, the generated hand channel profile in session 5 is very similar (in fPCA feature space) to a hand channel profile generated during session 1. (B) Analyses are as in A, but using the shin channel profile. The three selected fPCA components explained 85.8% of the variance in shin channel profiles across all participants and no-feedback trial conditions.* |
