# Supplementary material for: De novo sensorimotor learning through reuse of movement components
Source: PLoS Comput Biol. 2024 Oct 10;20(10):e1012492. doi: 10.1371/journal.pcbi.1012492 (PMC11495618; doi:10.1371/journal.pcbi.1012492)
Supplement: Attachment — Submitted filename: response-to-reviewer-comments.docx [file pcbi.1012492.s008.docx]

Dear Dr Haar,

We thank the editorial team and the reviewers for considering our manuscript (PCOMPBIOL-D-24-00867) entitled “De Novo Learning Through Reuse of Movement Components”.

In response to the reviewers’ comments, we have made several changes to clarify the key points of the work. In brief, we have addressed the recommendations of Reviewer 1 by making our data publicly available via an OSF repository and providing a video example of our main empirical task. We have also addressed the recommendations of Reviewer 2 by adding a more detailed justification of our methodological choices to the methods and discussion sections. We believe that the concerns raised by Reviewer 3 were not due to inherent weaknesses in our study, but were instead due to insufficient clarity in our reporting of methods and analyses. We have now clarified our analyses with additional description and justification, and have added a new supplementary analysis to more strongly demonstrate our key findings about reuse of motor outputs across sessions and conditions.

We give a detailed response to each of the reviewers’ comments below.

Yours sincerely,

George Gabriel

# Responses to Reviewer Comments

## Reviewer 1

Reviewer 1 stated no concerns, and made one recommendation:

*“…include a link to a video demo of the task, showing the cursor and target trajectories during the training and test trials.”*

We have created a video of the main path-following task, including an example trial of the main task and a video schematic showing the transformation of smoothed EMG into cursor movement. The video's URL is directly linked in our paper, and the file can also be accessed through the OSF repository (now also linked in the “Data Availability” section).

## Reviewer 2

### Major Comments:

Reviewer 2 provided two major comments:

#### Comment 1

*“A lot of interesting and important material appears only as supplementary materials… I would suggest that a lot or at least some of this be incorporated into the main figures in the paper.”*

We agree with the reviewer’s observation and have moved several figures from the supplementary material into the main text. The main figures now describe the methodology and the main conclusions of the study more fully. Specifically, we have moved former figures S2 (example cursor trajectories), S3 (transfer of performance improvements) and S6 (channel peak timing) to the relevant sections of the main text.

#### Comment 2

*“The choice to have participants perform the task without online visual feedback is slightly surprising. It's not clear why this was done… This requires a bit more justification and discussion of the implications of this choice.”*

To address this comment, we have added a clarifying explanation of this design choice and a discussion of its implications for our conclusions about reuse of motor commands. The final paragraph of “Free Movement and Path Following Tasks” in our Methods section extends our description of the task protocol by explicitly justifying our choice to not use online visual feedback. In brief, our justification is that the type of feedback provided to participants needed to be consistent between training and test blocks, to minimise the likelihood that participants would use different strategies to complete the path-following tasks in these two conditions. However, using online visual feedback during test blocks is not viable, as this could induce learning on the untrained paths, confounding our arguments about generalisation. Our choice to use post-trial visual feedback and to only include it in training trials overcomes both issues. We have now made this point explicit in the methods section of the paper.

We have also extended the discussion with a new section to clarify the scientific implications of our choice to limit visual feedback. In particular, we discuss how this decision may have limited performance improvement over the training sessions. We also observe that the absence of online visual feedback may have influenced the type of controller learned by the participants, as suggested by previous research^[1]^. We suggest that our finding about reuse of pre-existing motor commands may be limited to tasks in which a feedforward controller is used instead of a feedback controller, and point to further research which may help to test this possibility.

### Minor Comments:

Reviewer 2 also provided two minor comments:

#### Comment 1

*“Telgen, Parvin, Diedrichsen, ( J Neurosci 2014) ought to be cited somewhere as conceptually a very important paper…”*

We have now added this citation to our introduction section.

#### Comment 2

“In the Introduction: "Secondly, the participant may learn a new association between task goals and existing motor commands (Golub et al., 2018) ... Thirdly, the participant may have to learn to reliably and rapidly select task-appropriate commands from their existing repertoire. " It's not clear to me exactly what the difference is between these two ideas…”

We have rewritten the relevant section of the introduction to clarify these concepts. In brief, we distinguish between learning entirely new commands, learning that existing commands are suitable for achieving a given goal, and learning to more reliably and rapidly generate a command which is known to be suitable for a given goal. We accept the reviewer’s observation that learning an “association”, in the classic sense of the associative stage of learning, is not clearly distinguished from learning “to rapidly select” motor commands, and we have removed this terminology to avoid confusion.

## Reviewer 3

We are grateful for the detailed comments provided by reviewer 3, and have thoroughly considered the issues raised. We have directly implemented several of the reviewer’s suggestions, and have clarified the explanation of our results and methodology to address the remaining comments.

### Major Comments:

Reviewer 3 raised four major comments:

#### Comment 1

*“The use of Bayes factor instead of more common statistical methods like ANOVA (assuming normal distribution of the data) is a major weakness. The authors should justify their choice and possibly include traditional statistical analyses to strengthen their findings.”*

We agree that traditional hypothesis testing techniques such as ANOVA remain more common than Bayesian analyses in this field of research. This relative unfamiliarity of Bayesian methods may make our paper less accessible to some readers. To reduce this possibility, we have added additional guidance to the Data Analysis section describing how to interpret Bayes factors and our Bayesian models more generally.

To directly justify our choice of Bayesian analysis methods, we have also included a detailed description of the benefits of Bayes Factors over traditional hypothesis testing methods for our specific use case. Most notably, our Bayes factors do not require restrictive distributional assumptions (particularly relating to homogeneity of variance) which are not met by our data^[2]^. These points are argued in more detail in our amended Methods section. For the interested reader, we also cite a key source describing the benefits of Bayesian analysis in psychological studies.

#### Comment 2

*“The protocol included three train-test mini-sessions per day over five days, with five test trials per session. This might have led to learning during the test trials, confounding the generalization results. A more careful analysis is required to address this potential issue.”*

We are grateful for this thought-provoking comment and have considered a range of ways to address it through further analyses. After much consideration, we have concluded that analysis of learning during the test trials is not possible using our data. However, we believe that the design of our study and the results of our existing analyses imply that learning during test trials was limited, if present at all. Our arguments are summarised below.

We interpret the reviewer’s main concern to be that the apparent reuse of muscle activity patterns (termed “profiles” in the paper) in the trained and untrained conditions could be due to direct learning of the untrained conditions during test trials. The purported reuse of muscle activity patterns would then be a coincidence, with the same patterns being learned in both cases perhaps simply because they were useful in both cases. This idea is inconsistent with our results. In particular, we observed that participants reuse muscle activity patterns from R1 (R3) to follow L1 (L3) despite those patterns not being useful for the leftward paths (see Figure 5C in the revised paper). Even when these muscle activity patterns resulted in low error for the trained rightward conditions, they would often result in high error for the untrained leftward conditions of equal magnitude. If the participants had directly learned appropriate muscle activity patterns for the untrained conditions, they should not have learned patterns which so closely matched those used in the rightward conditions. Our results are therefore consistent with reuse of learning from the trained conditions in the untrained conditions, despite these motor commands being relatively ineffective for the latter conditions.

The design of our study provides further confidence that learning during the test trials should be limited. The design of our study was such that the only ways to improve performance on the untrained conditions (except through random exploration) depended upon transfer or generalisation of learning from the trained conditions. As the R2, L1, L2, and L3 paths were not practised with post-trial feedback, there was no opportunity for participants to directly learn motor outputs suited to these paths using visual feedback. Other sources of sensory input, such as tactile or proprioceptive input, do not provide information about cursor velocity or position, except if an association has already been learned between these sources of input and the cursor kinematics. Such an association can only be learned from visual feedback, because cursor position was only represented visually during this study. As visual feedback is available for only the trained conditions, such an association can only be directly learned for those conditions. Learning from non-visual sensory feedback in the untrained conditions therefore requires transfer of learning from the trained conditions.

We also note that opportunities for practice of the untrained conditions were very limited compared to the trained conditions. The untrained paths were encountered on far fewer trials (10 per training block, totalling 60 per day) than the trained ones (30 per training block, and 10 per test block, totalling 180 trials with feedback and 60 without feedback per day). As we observed only limited (but statistically robust) performance improvements on the conditions trained most often and with visual feedback, we expect that any direct learning on the conditions encountered less often and without visual feedback would be very limited.

Finally, we suggest that further analyses of learning during the no-feedback trials are not viable for two main reasons. Firstly, participants completed only 10 trials per condition in the test blocks. This limited amount of data, combined with the high variability in participant behaviour, severely limits our ability to detect trends in performance during a single test block. This observation was a key motivation for our use of session-averaged data to detect trends in error and variability. Secondly, the test blocks were always interspersed with training blocks in which participants received feedback on their execution of R1 and R3 condition trials. This makes performance trends across multiple test blocks unsuitable for identifying learning, as the interspersed training blocks would make it impossible to determine whether any observed performance changes between test blocks were due to generalisation from the training blocks or direct learning on the test blocks.

In conclusion, while we accept the reviewer’s suggestion that learning could have occurred during the test trials, we believe that any such learning would have been severely limited. This view is further supported by the observation that idiosyncratic muscle activity patterns from the trained conditions were reused in the untrained conditions, despite resulting in higher error in the untrained conditions.

#### Comments 3 and 4

*“(3) I believe that a control experiment to test the re-use hypothesis is missing. To verify if participants indeed produced the optimal pattern needed to succeed in the task already from Day 1, a new group should be tested but learn different pathways (e.g., R2 and L2) and then be examined for generalization and improvement to test the re-use hypothesis.”*

*“(4) Figure 4- The correlation between peak time and amplitude might be achieved through various means (since a point from each measure was selected). Precise comparison of the entire path as done with RMS error is needed. This needs further clarification and analysis”*

We thank the reviewer for these suggestions. After detailed consideration, we believe that comments 3 and 4 both stem from to a lack of clarity in our reporting of the analysis methods used in Figure 4. We have clarified these points in the paper, and added new supplementary analyses to address the reviewer’s concerns. We directly respond to the reviewer’s comments below.

Comment 4 appears to interpret former Figure 4 (now Figure 6) as showing a correlation between channel peak time and amplitude. In fact, we intended this figure to show similarity (distance of points from the diagonal) rather than correlation. It demonstrates the strong and consistent similarity between channel amplitudes (subfigure A) or peak times (subfigure B) in session 5 (horizontal axis) and session 1 (vertical axis). For each R1 and R3 trial in session 5, we computed the absolute difference between the channel amplitude (or peak time) on that trail and all no-feedback trials in session 1. For each session 5 trial, we retained the minimum of these differences. From the set of these minimal distances, we then selected the 99^th^ percentile value for each participant. We then plotted the corresponding session 5 value against the session 1 value for each participant. The distance of the plotted points from the diagonal is very small in most cases. This shows that 99% of the trials in session five involved channel peak amplitudes (and peak times) which were very similar to those generated on at least one of the no-feedback trials in session 1. We interpreted this as showing that per-channel outputs generated in session 5 could already be generated by most participants in session 1.

We are aware that the analysis method used to produce this figure is unconventional, but we believe it is justified by the directness with which the resulting figure demonstrates the above-stated point about reuse. To avoid misinterpretation, we have rephrased the figure caption with a more careful description of this methodology and guidance on the proper interpretation of the figure.

Comment 4 also observes a limitation relevant to the above-described analysis: it only considers the peak amplitude and timing of the generated muscle activity profiles, but muscle activity profiles are not uniquely determined by these two features. To strengthen our claim about reuse, we have added an additional analysis using functional principal component (fPCA) analysis. Using fPCA, we transformed each trial’s muscle activity profile into a three-dimensional feature-space representation. These three dimensions capture >85% of the variance in hand and shin muscle activity profiles across all participants and trials. We then plot the same similarity comparison figures described above for each of the feature dimensions, observing qualitatively similar results for each dimension. This analysis strengthens our claim that, in session 1, participants could already generate muscle activity profiles closely resembling those observed in session 5.

Comment 3 suggests an additional experiment to verify that participants “produced the optimal pattern needed to succeed in the task already from Day 1”. We note that our intention in Figure 4 and our new fPCA analysis was not to demonstrate that participants generated an “optimal” pattern. Rather, we attempted to show that, while participants’ performance improved on the trained tasks between sessions 1 and 5, these improvements in performance were not associated with learning to generate novel per-channel motor outputs. We believe that our new similarity analyses demonstrate this very clearly.

The reviewer’s suggested control experiment using different trained paths would test whether this finding was specific to our choice of trained paths (R1 and R3). However, our results already suggest that reuse from session 1 to session 5 is not specific to the trained paths. To show this more clearly, we have added new supplementary figures (S6 and S7) which repeat the similarity analysis for the untrained path conditions. We observe that reuse across sessions is observed in these untrained conditions too.

Our main conclusion is that novel coordinated control tasks can be learned by reusing existing motor commands. Based on the above arguments, we believe that our existing results (accompanied by new supplementary analyses and clearer explanations) are sufficient to justify this conclusion. While we agree that an additional training study with different trained paths would further strengthen our conclusion, we believe that the value added by such a study would be limited. Instead, we suggest that future studies could more robustly test our findings by using different tasks or different interfaces, to assess the general conditions in which the reuse of existing motor commands can support the learning of a new coordinated control task.

### Minor Comments:

Reviewer 3 also raised four minor comments.

#### Comment 1

*“The phrase "Such skills cannot be learned by adjusting an existing control policy" in the Significant Statement is an overstatement. It should be tempered and rewritten for accuracy.”*

We have rewritten this part of the significance statement to improve its clarity and accuracy.

#### Comment 2

*“Add the experimental protocol to Figure 1 for clarity.”*

We have added a diagram showing the block structure of the main experiment.

#### Comment 3

*“Page 18, line 367: The text should indicate "reduced error" instead of "increased accuracy".”*

We have changed this wording as suggested.

#### Comment 4

“Page 33, line 435: The phrase "...whose trajectory shapes did not improve..." is inaccurate as some participants did improve. Figure 3B also needs clarification regarding the classification of participants who did not improve but aligned with the correlation line.”

We have added a legend to clarify that former Figure 3B (now figure 5B) shows participants grouped as improvers and non-improvers based on RMS error, rather than the congruent/incongruent grouping used elsewhere. To clarify how we defined improvers and non-improvers, we include a description of the Bayes factor-based threshold in the figure caption.

By participants “whose trajectory shapes did not improve”, we refer to participants who were classified as non-improvers based on their trajectory shape (or, more specifically, the RMS error therein).

We have also added an additional sentence to the main text relating to former Figure 3B to clarify that, while dissimilarity of hand and shin profiles is a sufficient condition for non-improvement in the L1 and L3 cursor trajectories, it is not a necessary condition.

1. Raichin, A., Rabani, A. S., & Shmuelof, L. (2021). Motor skill training without online visual feedback enhances feedforward control. Journal of Neurophysiology. https://doi.org/10.1152/jn.00145.2021
2. Several of our figures directly demonstrate heteroscedasticity of error and timing across sessions. These changes are made clear by the use of Bayes factors.
